# Bilateral Cranial Nerve VI Palsies in Cryptococcal Meningitis, HIV, and Syphilis: A Case Report

**DOI:** 10.5811/cpcem.2021.8.53347

**Published:** 2021-11-01

**Authors:** Germaine Rival, Onyi Okorji, Rachael Kern, Preya Patel, Kate Fradeneck, Darragh Cullen

**Affiliations:** Jefferson Health Northeast, Department of Emergency Medicine, Philadelphia, Pennsylvania

**Keywords:** bilateral cranial nerve VI palsies, cryptococcal meningitis, HIV/AIDS, syphilis, case report

## Abstract

**Introduction:**

Cranial nerve (CN) VI palsy is a common complaint seen in the emergency department (ED) and has a wide range of causes. Bilateral CN VI palsies are uncommon and appear to be associated with more severe complications.

**Case Report:**

A 29-year-old male presented to the ED from an ophthalmology office for diplopia, headache, and strabismus. He was found to have bilateral CN VI palsies and new-onset seizure in the ED. A lumbar puncture revealed cryptococcal meningitis. Additional tests revealed a new diagnosis of human immunodeficiency virus (HIV), acquired immunodeficiency syndrome (AIDS), and syphilis.

**Conclusion:**

Cryptococcal meningitis remains a life-threatening complication of HIV/AIDS. Coinfections with HIV, particularly syphilis, further complicate a patient’s prognosis as both can lead to devastating neurological sequelae. In cryptococcal meningitis, elevated intracranial pressure is a complication that can manifest as seizures, altered mental status, and cranial nerve palsies.

## INTRODUCTION

Cranial nerve (CN) palsies involving the third, fourth, and sixth cranial nerves are common complaints seen in the emergency department (ED). Cranial nerve VI palsy is the most common isolated ocular neuropathy and is seen in 50% of patients.^1^ Causes of CN VI palsies include trauma, demyelination, neoplasm, microvascular ischemia, infection, and elevated intracranial pressure (ICP).^1^ Bilateral CN VI palsies herald ominous causes, such as intracranial hemorrhage and brainstem infarction.^1^ Patients with this palsy typically present with the inability to abduct the affected eye. Given the potentially life-threatening implications associated with bilateral CN VI palsy, it is important to determine the cause and manage it immediately.

## CASE REPORT

A 29-year-old male with a past medical history of Kawasaki disease presented to the ED from an outpatient ophthalmology office for diplopia and strabismus for three days. The patient reported frontal headache with rhinorrhea for five days and was being treated with clindamycin for suspected sinusitis by his primary care physician. The patient was subsequently evaluated by an ophthalmologist, who diagnosed him with bilateral cranial nerve VI palsies and referred him to the ED for further evaluation and management. The patient denied fever, chills, slurred speech, facial droop, vision loss, weakness, or recent trauma. The patient also denied any surgical history, medications, allergies, or any history of smoking, excessive alcohol use, or recreational drug use. While in the ED, the patient had a witnessed tonic-clonic seizure, which was successfully managed with intravenous (IV) lorazepam.

**Image 1:**
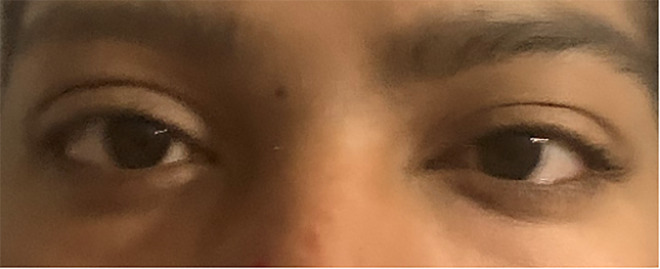
Disconjugate gaze of the left eye.

**Image 2:**
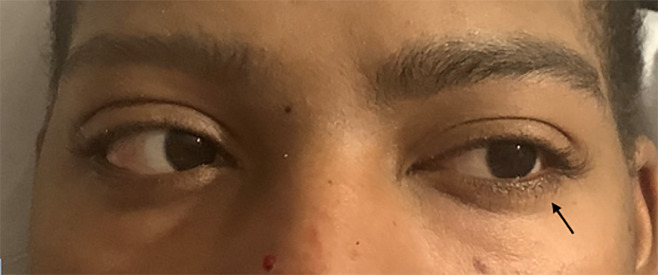
Leftward gaze. Patient with difficulty abducting left eye with a leftward gaze.

**Image 3:**
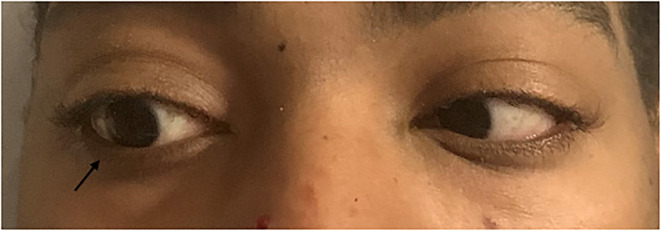
Rightward gaze. Patient with difficulty abducting right eye with a rightward gaze.

On exam, his vitals were as follows: temperature of 37.1 °C, blood pressure 142/88 millimeters mercury, heart rate 94 beats per minute, respiratory rate 20 breaths per minute, and pulse oximetry 100% on room air. The patient was well-appearing and in no acute distress. His head, eyes, ear, nose, and throat exam showed round and equally reactive pupils without nystagmus. The patient’s neurologic exam showed an awake, alert and oriented male with bilateral CN VI palsies. Apart from his bilateral CN VI palsies, the rest of his cranial nerve exam was grossly intact. He had 5/5 muscle strength in upper and lower extremities. Finger to nose test was intact. His skin exam revealed bilateral palmar rashes (Images 1, 2, 3).

The patient’s blood tests were significant for mild hyponatremia, leukopenia, and lymphopenia. Computed tomography (CT) of his brain without IV contrast revealed no mass or hemorrhage. Magnetic resonance imaging (MRI) of his brain and cranial nerves were likewise unrevealing for acute pathology.

**CPC-EM Capsule**

What do we already know about this clinical entity?

*Cryptococcal meningitis is a well-known complication of HIV/AIDS, which can present with unilateral cranial nerve palsy. However, literature on bilateral cranial nerve palsies is limited.*

What makes this presentation of disease reportable?

*This patient presented with atypical symptoms of bilateral CN VI palsies associated with new diagnoses of cryptococcal meningitis, HIV, AIDS, and syphilis.*

What is the major learning point?

*Bilateral CN VI palsies are atypical, yet critical presentations of cryptococcal meningitis, HIV/AIDS, and syphilis.*

How might this improve emergency medicine practice?

*Recognizing atypical presentations of cryptococcal meningitis, such as bilateral CN VI palsies can lead to early evaluation, diagnosis and management.*

He was subsequently admitted for further investigation of his diplopia, bilateral CN VI palsies, and new-onset seizure. Further inpatient evaluation involved a CT venography of the brain, which showed no evidence of cavernous sinus thrombosis. A lumbar puncture revealed an opening pressure of 60 centimeters of water (cm H_2_O) (reference range: 10–25 cm H_2_O), cerebrospinal fluid (CSF) glucose 28 milligrams per deciliter (mg/dL) (40–70 mg/dL), CSF protein 41 mg/dL (15–45 mg/dL), and a positive CSF cryptococcal antigen. Serum rapid plasma reagin and fluorescent treponemal antibody absorption test were both reactive. The fourth-generation HIV immunoassay revealed positive serum HIV-1 antigen and HIV-1 antibody. Absolute CD4 count was 6 cells per microliter (μL) (490 – 1740 cells/μL). In the presence of cryptococcal meningitis along with an absolute CD4 count of 6 cells/μL, the patient met diagnostic criteria for AIDS. The patient was treated with penicillin, amphotericin B, and flucytosine. Trimethoprim-sulfamethoxazole was administered as prophylactic treatment for Pneumocystis carinii pneumonia. The patient was discharged with anti-retroviral therapy and outpatient follow-up with infectious disease. He returned to the ED a few months later for altered mental status and was found to have recurrent cryptococcal meningitis and CN palsy.

## DISCUSSION

Cryptococcal meningitis is a widely recognized complication in immunocompromised patients, particularly those with solid-organ transplants as well as those with HIV/AIDS. It is the leading cause of mortality in these populations, and HIV remains the primary risk factor in contracting cryptococcus worldwide. Elevated ICP, defined as CSF pressure greater than 25 cm H2O, is a common complication of cryptococcal meningitis, which results from a failure of CSF resorption in the arachnoid villa due to physical obstruction by the cryptococcal polysaccharide capsule, thus leading to cerebral edema.^2^ The larger the size of the cryptococcal capsule and the amount of cryptococcal organisms present in the arachnoid granulations, the more elevated the ICP.^2^ Cerebral edema can manifest as headaches, persistent vomiting, papilledema, vision disturbances, blindness, cranial nerve palsy, altered mental status, and coma.

Diagnosis is made by microscopy with India ink staining of CSF or a Sabouraud dextrose agar culture. More recently, cryptococcal antigen flow assays have been used due to their high sensitivity and specificity. Despite this, it is important to note that a normal CSF analysis does not exclude cryptococcal meningitis; this is, in fact, a poor prognostic indicator.^3^ According to the 2010 Infectious Diseases Society of America guide-lines, a combination of amphotericin B and flucytosine is the recommended antifungal regimen for cryptococcal meningitis in HIV patients. Management of cryptococcal meningitis can be categorized into three phases: 1) induction; 2) consolidation; and 3) maintenance therapy.^3^ Induction therapy’s aim is rapid clearance of cryptococcus in the CSF. This can be achieved with a combination of amphotericin B 0.7–1.0 (milligrams per kilogram per day (mg/kg/day) intravenously and flucytosine 100 mg/kg daily orally. Consolidation therapy should be initiated approximately two weeks after induction therapy and consists of fluconazole 400 mg/day for at least eight weeks. After successful sterilization of the CSF with induction and consolidation therapies, culture-negative patients can transition to fluconazole 200 mg/day for maintenance therapy.^4^

Syphilis, a sexually transmitted infection caused by Treponema pallidum, has been resurgent in the United States over the past 20 years. Furthermore, high rates of coinfection between syphilis and HIV have been found, which has been largely attributed to similar at-risk populations, such as men who have sex with men.^5^ Studies have also demonstrated that HIV viral load is higher when patients are coinfected with syphilis when compared to those without this coinfection. Moreover, HIV patients whose syphilis infections have been treated have shown decreased HIV viral loads.^6^ Therefore, it is crucial to screen for additional sexually transmitted infections, particularly syphilis, when a diagnosis of HIV/AIDS is suspected or established. Intramuscular penicillin is the recommended antibiotic treatment for syphilis.

Isolated cranial nerve involvement in cryptococcal meningitis is well-documented in literature. However, literature involving bilateral CN VI palsies have been sparse. Case reports of CN VI palsy from infectious causes include Lyme disease, varicella zoster virus (VZV) and neurosyphilis. Nevertheless, these cases are often unilateral and involve additional cranial nerves, such as oculomotor, facial and vagus nerves.^7^, ^8^, ^9^ Joo, Lee and Kim (2019) reported a 71-year-old female who presented with vocal cord paralysis and diplopia who later developed a skin rash. A biopsy of the skin lesions showed VZV and the mechanism by which CN VI was involved remain poorly understood.^7^ Another case report involving unilateral CN VI palsy was discussed by Lundin et al (2020) in a 70-year-old man with right eye pain and double vision who later developed erythema migrans. This patient was found to have optic neuritis with CN VI palsy secondary to Lyme disease.^8^ Jordan, Marino and Damast (1978) were one of the first to document a case report of bilateral cranial nerve paresis in a 61-year-old male with neurosyphilis; however, it involved the oculomotor nerves (CN III) only.^9^

Although this patient was also diagnosed with syphilis, which could have contributed to his bilateral CN VI palsies, his symptoms appeared to improve once amphotericin B and flucytosine were initiated to treat his cryptococcal meningitis, suggesting a possible association between cryptococcal meningitis and CN VI palsy. This could perhaps be due to improvement in cerebral edema and ICP as treatment for meningitis was continued. This case report highlights the possible association of elevated ICP in cryptococcal meningitis with bilateral CN VI palsies complicated by a coinfection with HIV/AIDS and syphilis. Therefore, it is important to stress the significance of immediate recognition and management of cryptococcal meningitis, especially in patients exhibiting signs of increased ICP.

## CONCLUSION

Sexually transmitted infections and complications of HIV/AIDS are common complaints seen in the ED today. Cryptococcal meningitis is a common yet life-threatening complication of HIV. A patient’s prognosis could be further complicated by the presence of coinfections, particularly syphilis as both infections have been associated with severe neurological sequelae. Therefore, it is important for emergency physicians to remain vigilant and maintain a high index of suspicion when managing patients with known HIV infection or with atypical presentations.
